# Role of peptide transporters in small peptide uptake of bovine mammary epithelial cells cultured in a transwell chamber

**DOI:** 10.1002/fsn3.3343

**Published:** 2023-03-31

**Authors:** Miaomiao Zhou, Fei Huang, Yehui Qi

**Affiliations:** ^1^ College of Agricultural Science and Engineering Liaocheng University Liaocheng P.R. China

**Keywords:** bovine mammary epithelial cells, small peptide transport, transwell, β‐Casein

## Abstract

Small peptides can be absorbed by the bovine mammary gland for the synthesis of milk protein, but the absorption mechanism still needs further study. In this study, the role of peptide transporters in small peptide uptake by bovine mammary epithelial cells (BMECs) was studied. First, BMECs were obtained and cultured in a transwell chamber. After 5 days of culture, the FITC‐dextran permeability of the cell layer was detected. Then, 0.5 mM methionyl‐methionine (Met‐Met) was added to the medium of the lower and upper transwell chambers, respectively. The culture medium and BMECs were collected after 24 h of treatment. Liquid chromatography‐mass spectrometry (LC–MS) was used to detect the concentration of Met‐Met in the culture medium. Real‐time PCR was used to detect the mRNA abundance of β‐casein, oligopeptide transporter 2 (PepT2), and small peptide histidine transporter 1 (PhT1) in BMECs. Then, the BMECs were transfected with siRNA‐PepT2 and siRNA‐PhT1, respectively, and the uptake of β‐Ala‐Lys‐N‐7‐amino‐4‐methylcoumarin‐3‐acetic acid (β‐Ala‐Lys‐AMCA) in BMECs was detected. The results showed that, after 5 days of culture, the FITC‐dextran permeability of BMECs was 0.6%, which was significantly lower than that of the control group. The absorption rates of Met‐Met in the culture medium of the upper and lower chambers were 99.99% and 99.95%, respectively. The addition of Met‐Met to the upper chamber significantly increased the mRNA abundance of β‐casein and PepT2. The addition of Met‐Met to the lower chamber significantly improved the mRNA abundance of β‐casein, PepT2, and PhT1. The uptake of β‐Ala‐Lys‐AMCA significantly decreased in BMECs transfected with siRNA‐PepT2. These results suggested that the BMECs were successfully cultured in the transwell chamber and formed a cell layer with little permeability. The small peptides in both the upper and lower chambers of the transwell can be absorbed by BMECs in different ways. PepT2 plays an important role in the uptake of small peptides on both the basal and apical sides of BMECs, and PhT1 may be involved in the uptake of small peptides on the basal side of BMECs. Therefore, the addition of small peptides in dairy cow diets may be an effective dietary manipulation to increase milk protein concentration or yield.

## Graphical abstract

In this study, role of oligopeptide transporter 2 (PepT2) and small peptide histidine transporter 1 (PhT1) on small peptide uptake in bovine mammary epithelial cells (BMECs) cultured in transwell chamber was studied for the first time. The results suggest that the small peptides in both upper and lower chambers of transwell can be absorbed and used for milk protein synthesis by BMECs in different ways; PepT2 plays an important role in the uptake of small peptides in both basal and apical side of BMECs, and PhT1 may be involved in the uptake of small peptides in the basal side of BMECs.

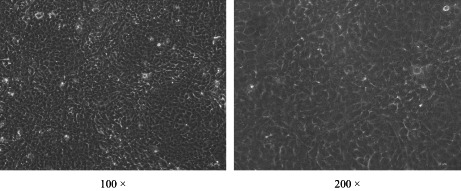

## 1 INTRODUCTION

Milk protein of dairy cows is an important source of protein for human beings. The milk protein includes casein and whey protein, of which casein accounts for 80%. The casein can be mainly divided into α_s1_‐casein (10 g/L), α_s2_‐casein (3.7 g/L), β‐casein (10 g/L), and κ‐casein (3.5 g/L) (Pastuszka et al., 2016). Milk protein synthesis in the bovine mammary gland is a complex biochemical process. Both free amino acids and small peptides can be taken up by the mammary gland for milk protein synthesis (Tagari et al., 2004; Zhou et al., 2021). Small peptides play a crucial role in the synthesis of milk proteins (Chen et al., 1987; Tagari et al., 2008). In addition to being used as a nutritional substrate, small peptides can promote milk protein synthesis by enhancing the absorption of amino acids and serving as signaling molecules (Chen et al., 2020; Zhou et al., 2021).

Small peptides can be transported into the bovine mammary gland in intact form by oligopeptide transporters. The known proton‐coupled oligopeptide transporters (POT) include oligopeptide transporter 1 (PepT1), PepT2, small peptide histidine transporter 1 (PhT1), and PhT2, which can transport oligopeptides and peptide analogs to various cells in reverse concentrations driven by electron gradients (Fowler et al., 2015; Smith et al., 2013; Verri et al., 2017); they play important physiological roles in animal growth and metabolism (Alghamdi et al., 2019; Shimizu et al., 2004). Currently, research on POT mainly focuses on intestinal small peptide absorption and renal tubular reabsorption, as well as drug transport in the animal body (Daniel, 2004; Newstead, 2017; Oppermann et al., 2019). There are few studies on the role of POT in small peptide transport in bovine mammary epithelial cells (BMECs). It is confirmed that PepT2 and PhT1 are expressed and play a role in BMECs, and PepT1 and PhT2 are not expressed in bovine mammary glands (Wang et al., 2018; Zhou et al., 2011). However, whether PepT2 and PhT1 play a role in the mammary gland uptake of small peptides from the blood (basement membrane side of BMECs) still needs further study. In addition, it is found that the fluorophore‐conjugated dipeptides, β‐Ala‐Lys‐N‐7‐amino‐4‐methylcoumarin‐3‐acetic acid (β‐Ala‐Lys‐AMCA), can be used as hydrolysis‐resistant reporters which can be utilized to visualize and measure the activity of POT (Alghamdi et al., 2018). Therefore, the role of PepT2 and PhT1 in Met‐Met and β‐Ala‐Lys‐AMCA uptake of BMECs cultured in transwell chambers was studied. Furthermore, β‐casein is a major milk protein, accounting for more than 36% of the total casein. Thus, β‐casein was selected as the detection index in this study.

## 2 MATERIALS AND METHODS

### 2.1 Preparation and transwell culture of BMECs

Mammary tissues were obtained from three healthy mid‐lactation Chinese Holstein dairy cows. Then, the BMECs were isolated and purified according to the method of Zhou et al. (2015). The primary cultured cells were seeded at a density of 2 × 10^4^ cells/mL on the polycarbonate membrane of the transwell in 12‐well plates, and 0.5 mL and 1.5 mL DMEM‐F12 medium (supplemented with 1% glutamine, 100 IU/mL penicillin, 100 μg/mL streptomycin, 5 μg/mL insulin, 5 μg/mL prolactin, 1 μg/mL hydrocortisone, and 10% fetal calf serum) were added to the upper chamber and lower chamber, respectively. The cells were cultured at 37°C and the medium was changed every other day.

The cells of passages 4–5 were used in this study. After cells cultured in transwells formed a cell layer, the tightness of the cell layer was detected by evaluating the FITC‐dextran 4000 permeability. The treatment was divided into a cell‐free group (Control) and cell layer group (BMECs) with three repetitions each. The cells were washed three times with PBS, then 1.5 mL of 100 μM FITC‐dextran 4000 was added to the lower chamber, and 0.5 mL of medium was added to the upper chamber. The cells were incubated at 37°C for 2 h. At the end of incubation, 200 μL of upper chamber culture medium was taken, and the fluorescence value was measured by fluorescence microplate reader.

### 2.2 Liquid chromatography‐mass spectrometry (LC–MS)

The concentration of Met‐Met in the cell culture medium was determined by SCIEX Triple Quad 6500^+^ LC–MS/MS (SCIEX, USA). First, the samples were pretreated to remove impurities and extract Met‐Met: acetonitrile (400 μL) was added to the cell culture medium (100 μL), stirred for 1 min, and then centrifuged at 15,000× *g* for 10 min; the supernatant (400 μL) was concentrated and evaporated, 150 μL of 50% acetonitrile aqueous solution was added to redissolved the solute, and then centrifuged at 15,000× *g* for 10 min; and the supernatant was used for analysis. The LC–MS MRM mode was used to detect and collect the chromatographic information of Met‐Met. Then, MultiQuant™ software (SCIEX, USA) was used for Met‐Met data analysis. After obtaining the mass spectrum peak area of the Met‐Met sample, the concentration of Met‐Met in the cell culture medium was calculated according to the Met‐Met standard solution.

Chromatographic conditions: the chromatographic column was Waters Xbridge Amide (4.6 × 100 mm, 3.5 μm), aqueous phase A was water (containing 0.1% formic acid), organic phase B was acetonitrile (containing 0.1% formic acid), and the running time, injection volume, and column temperature were 12 min, 5 μL, and 35°C, respectively. The elution gradient is shown in Table 1.

**TABLE 1:** Elution gradient in chromatographic for separation of Met‐Met.

Time (min)	Flow rate (mL/min)	*B* (%)	Time (min)
0	0.4	60	0
1	0.4	60	1
6	0.4	25	6
8	0.4	25	8
8.01	0.4	60	8.01
12	0.4	60	12

Mass spectrometry conditions: positive ion (ESI+) MRM scanning mode was used. The specific mass spectrum parameters are shown in Tables 2 and 3.

**TABLE 2:** Mass spectrum parameters for Met‐Met chromatographic information detection and collection.

Mass spectrum parameters	MRM (ESI+)
ISV	5500 V
CUR	35 psi
TEM	550°C
GS1	55 psi
GS2	55 psi

**TABLE 3:** MRM ion pair, CE, and DP parameters for Met‐Met chromatographic information detection and collection.

Compound	MRM transition	Adduct ion	DP	CE
Met‐Met	281.3 → 104.1	[M + H]+	60	12

### 2.3 Real‐time PCR

Total RNA was extracted from BMECs with TRIzol® Reagent (Invitrogen, USA) and first‐strand cDNA was synthesized with the 5X All‐In‐One RT MasterMix kit (Abm, Canada). The relative mRNA abundance of β‐casein, PepT2, and PhT1 normalized to glyceraldehyde‐3‐phosphate dehydrogenase (GAPDH) was detected by real‐time PCR according to the method of Zhou et al. (2015). The primers for β‐casein, PepT2, PhT1, and GAPDH for real‐time PCR are shown in Table 4.

**TABLE 4:** Oligonucleotide primers of β‐casein, PepT2, PhT1, and GAPDH for real‐time PCR.

Gene	Forward primer (5′—3′)	Reverse primer (5′—3′)
β‐casein	AGTGAGGAACAGCAGCAAACAG	AGCAGAGGCAGAGGAAGGTG
PepT2	ATGGCAATGCCCAATGAAG	CACCAACACAGCAACAAACAAA
PhT1	GAGCTTTGTTACCGGCTACG	CGGCTTGGTGATGAAGAAGG
GAPDH	GCCAAGAGGGTCATCATCTC	GGTCATAAGTCCCTCCACGA

### 2.4 siRNA transient transfection

After BMECs in transwells reached 70–80% confluence, the cells were transfected with various siRNAs. The siRNA interference sequences and transfection method were performed according to Wang et al. (2019). The siRNA interference sequences are shown in Table 5. The nontransfected cells were used as the control group, and the cells transfected with empty vectors were used as the negative control group. Two days after transfection, the gene expression of PepT2 and PhT1 in the BMECs was determined, and the interference efficiency was calculated.

**TABLE 5:** The siRNA interference sequence for PhT1 and PepT2.

siRNA interference sequence	Sense (5′—3′)	Antisense (5′—3′)
Negative control (NC)	UUCUCCGAACGUGUCACGUTT	ACGUGACACGUUCGGAGAATT
siRNA‐PepT2‐1	CCAACUAUCCACUGAGUAUTT	AUACUCAGUGGAUAGUUGGTT
siRNA‐PepT2‐2	CCCUGUAUUUCCUGUAUUUTT	AAAUACAGGAAAUACAGGGTT
siRNA‐PepT2‐3	CCAUUGCUGACUCAUGGUTT	ACCAUGAGUCAGCAAUGGCTT
siRNA‐PhT1‐1	CCUACGAACGUCCGCAAUUTT	AAUUGCGGACGUUCGUAGGTT
siRNA‐PhT1‐2	CCAGCAGAACGUGAGCUUUTT	AAAGCUCACGUUCUGCUGGTT
siRNA‐PhT1‐3	CCCGGAAGAUAAAGUGGAATT	UUCCACUUUAUCUUCCGGGTT

### 2.5 Detection of β‐Ala‐Lys‐AMCA uptake

Transport studies of PepT2 and PhT1 in BMECs were determined using the β‐Ala‐Lys‐AMCA method (Alghamdi et al., 2018). After transfection, the BMECs were incubated in lower chamber medium with 50 μM β‐Ala‐Lys‐AMCA at 37°C for 20 min. The uptake of β‐Ala‐Lys‐AMCA was terminated by washing BMECs with ice‐cold PBS. Subsequently, the cells were lysed with 1% Triton‐100, and the amount of β‐Ala‐Lys‐AMCA taken up by cells was measured with a microplate reader (excitation at 350 nm and emission at 455 nm). The protein concentration was determined by the BCA method.

### 2.6 Statistical analysis

Data were analyzed using one‐way ANOVA and Duncan's multiple‐range tests using SAS software (SAS 9.2, SAS Institute Inc., Cary, NC). Values are shown as the means ± SDs. Bars with different superscripts are significantly different (*p* < .05).

## 3 RESULTS

### 3.1 Transwell culture of BMECs

**FIGURE 1:**
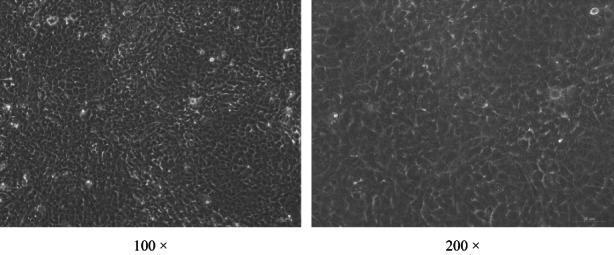
Bovine mammary epithelial cells cultured in vitro. Under the phase contrast microscope, the bovine mammary epithelial cells were pebble shaped and formed a cell layer. The magnifications are 100 times (left) and 200 times (right), respectively.

**FIGURE 2:**
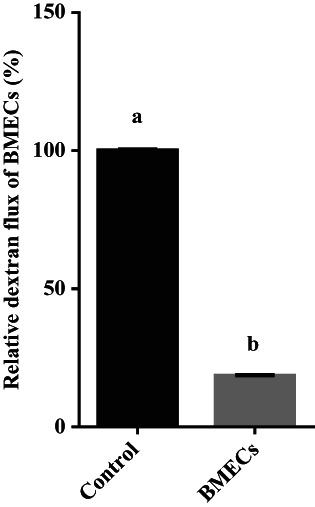
Permeability of FITC‐dextran in bovine mammary epithelial cell layer. After cells cultured in transwells formed a cell layer, the tightness of the cell layer was detected by evaluating FITC‐dextran 4000 permeability. The treatment was divided into a cell‐free group (control) and a cell layer group (BMECs). The FITC‐dextran permeability of BMECs group relative to control group was calculated. Values are means ± SD (*n* = 3). Bars with different superscripts are significantly different (*p <* .05).

BMECs were successfully obtained and cultured in the transwells (Figure 1). The permeability of FITC‐dextran in the cell layer was 18.6% of that of the control group (*p <* .05, Figure 2).

### 3.2 Absorption of Met‐Met by BMECs

**FIGURE 3:**
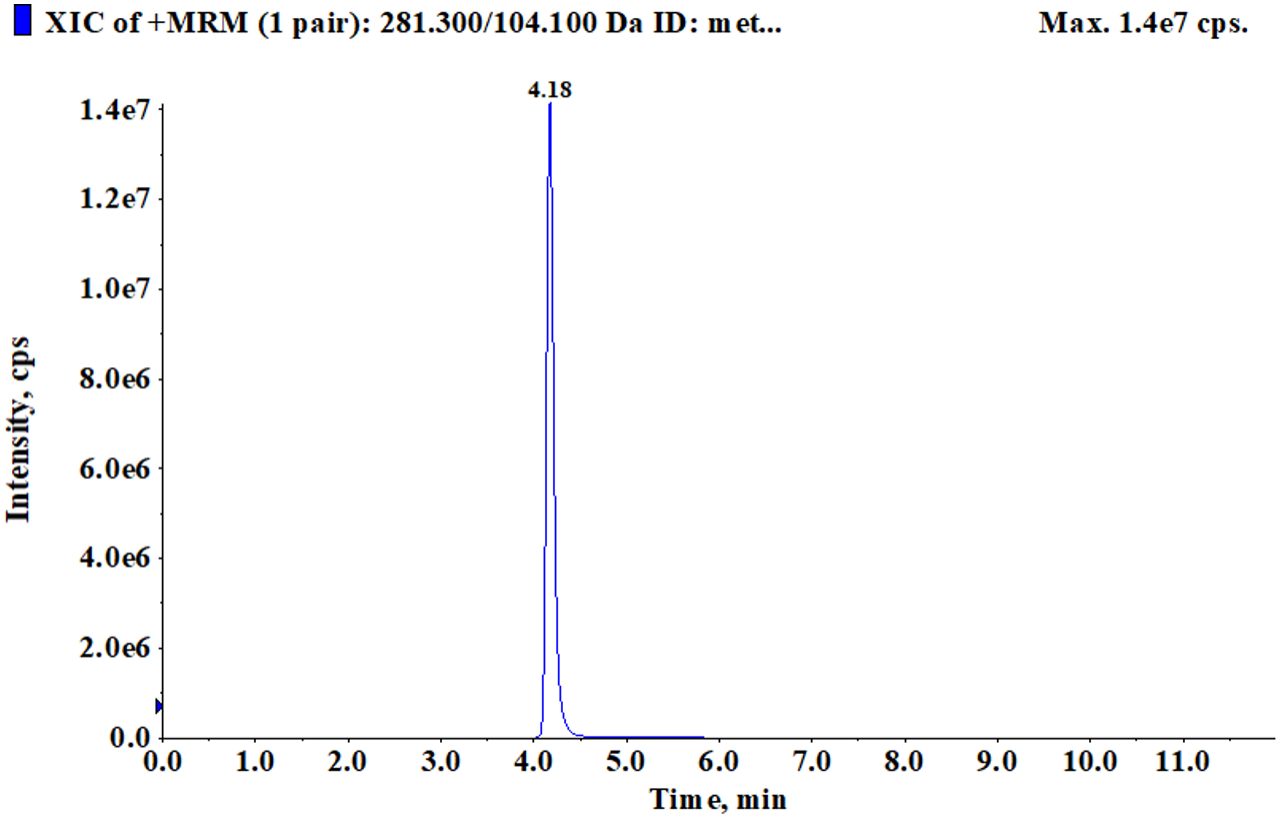
Typical chromatogram of methionine dipeptide. The LC–MS MRM mode was used to detect and collect the chromatographic information of Met‐Met. The retention time of Met‐Met was 4.18 min.

After BMECs formed a cell layer, 0.5 mM Met‐Met was added to the medium of the transwell upper and lower chambers, respectively. The culture medium was collected after 24 h of treatment. LC–MS was used to detect the concentration of Met‐Met. The retention time of Met‐Met was 4.18 min (Figure 3). The results showed that the concentration of Met‐Met in the upper chamber culture medium was 0.0052 ± 0.00023 μΜ, and that in the lower chamber culture medium was 0.2541 ± 0.020 μΜ (Table 6). The absorptivity of Met‐Met in the upper and lower chamber culture media was 99.99% and 99.95%, respectively.

**TABLE 6:** Concentration of Met‐Met in the culture medium of upper and lower chambers.

Culture medium	Concentration of met‐met (μΜ)	Average ± SD	Absorptivity of met‐met (%)
The upper chamber	0.005304489	0.0052 ± 0.00023	99.99
	0.004930996		
	0.005364515		
The lower chamber	0.252688746	0.2541 ± 0.020	99.95
	0.275194534		
	0.23441672		

### 3.3 Effects of Met‐Met on β‐casein, PepT2, and PhT1 mRNA abundance

**FIGURE 4:**
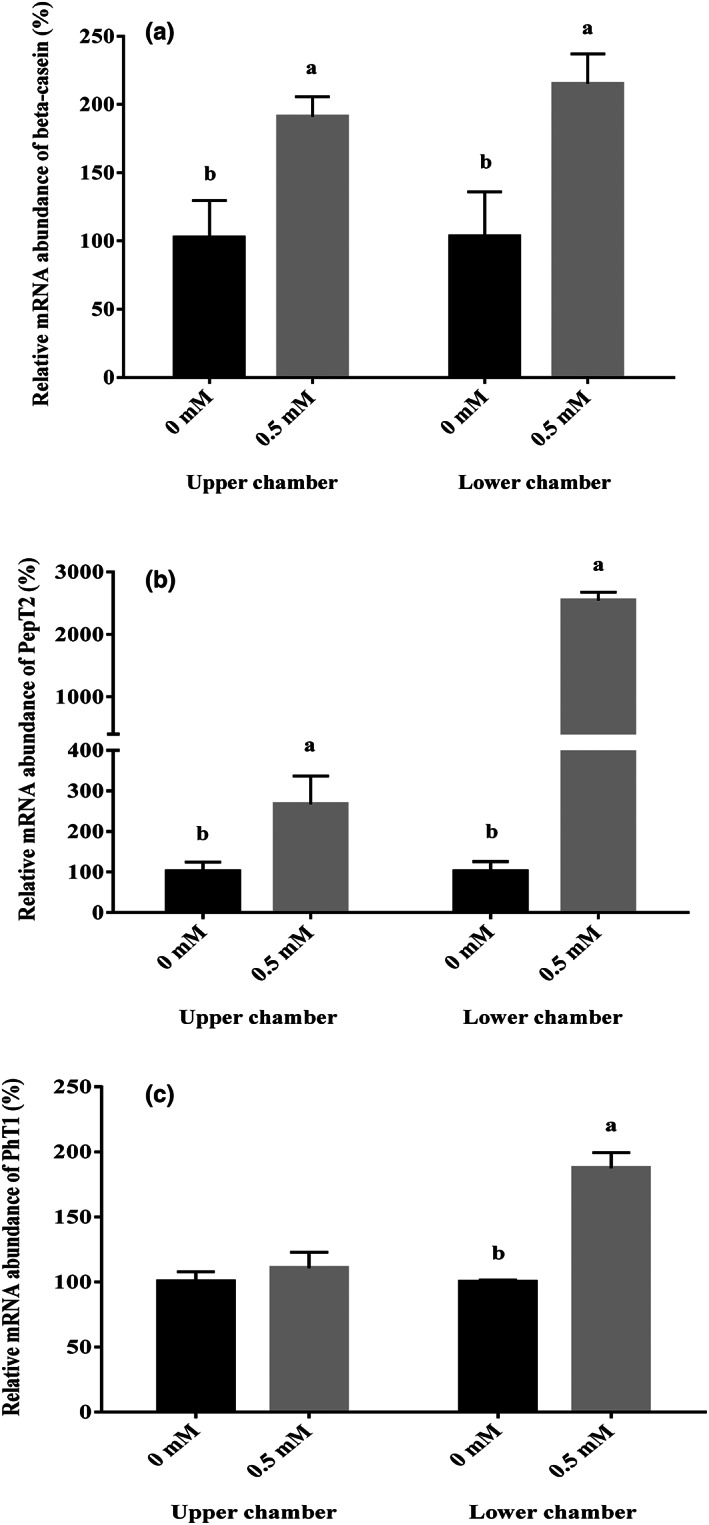
Effects of Met‐Met on β‐casein (a), PepT2 (b) and PhT1 (c) mRNA abundance. The genes mRNA abundance of experimental group (0.5 mM Met‐Met) relative to control group (0 mM Met‐Met) in upper or lower chamber was calculated, respectively. Values are means ± SD (*n* = 3). Bars with different superscripts are significantly different (*p <* .05).

After the formation of the BMEC cell layer, 0.5 mM Met‐Met was added to the upper and lower chamber culture media, respectively. The BMECs were collected after 24 h of treatment. Real‐time PCR was used to detect the mRNA abundance of β‐casein, PepT2, and PhT1. The results showed that the addition of 0.5 mM Met‐Met in both the upper and lower chambers significantly promoted the mRNA abundance of β‐casein and PepT2 (*p* < .05, Figure 4a,b). Met‐Met treatment in the lower chamber significantly increased the mRNA abundance of *PhT1* (*p <* .05, Figure 4c), and Met‐Met in the upper chamber had no effect on PhT1 gene expression (Figure 4c).

### 3.4 Effect of PepT2 interference on β‐Ala‐Lys‐AMCA uptake

**FIGURE 5:**
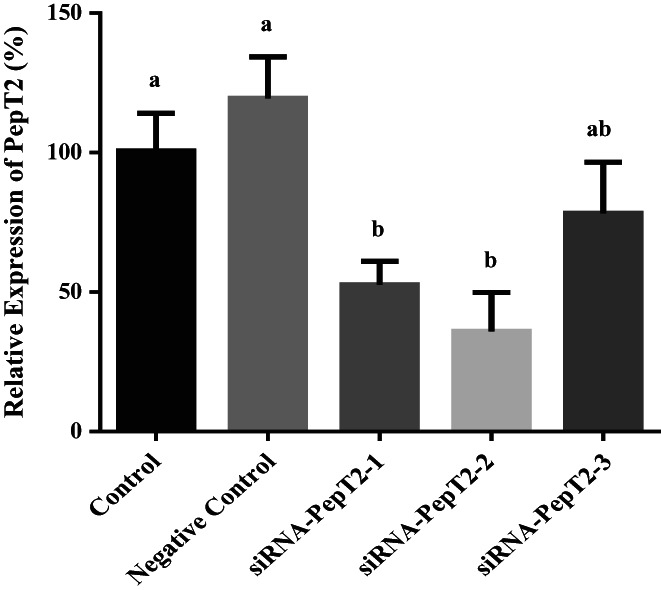
Effect of siRNA‐PepT2 on PepT2 mRNA abundance. The PepT2 mRNA abundance of siRNA‐PepT2s transfected groups (siRNA‐PepT2‐1, siRNA‐PepT2‐2, and siRNA‐PepT2‐3) and empty vectors transfected group (negative control) relative to nontransfected cells group (control) were calculated. Values are means ± SD (*n* = 3). Bars with different superscripts are significantly different (*p <* .05).

**FIGURE 6:**
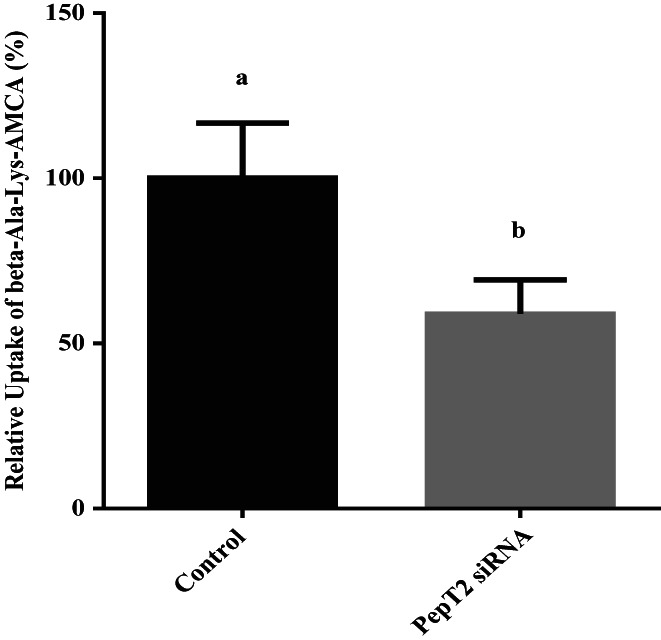
Effects of siRNA‐PepT2 on β‐Ala‐Lys‐AMCA uptake. The β‐Ala‐Lys‐AMCA uptake of siRNA‐PepT2 group relative to control group (nontransfected BMECs) was calculated. Values are means ± SD (*n* = 3). Bars with different superscripts are significantly different (*p <* .05).

The results showed that siRNA‐PepT2‐2 had the highest interference efficiency, 64%, and could be used in subsequent experiments (Figure 5). After transfection with siRNA‐PepT2‐2, the BMECs were incubated in lower chamber medium with 50 μM β‐Ala‐Lys‐AMCA at 37°C for 20 min. Then, the absorption of β‐Ala‐Lys‐AMCA by BMECs was detected. The results showed that after PepT2 interference, the uptake of β‐Ala‐Lys‐AMCA decreased by 41% (*p <* .05, Figure 6). These results suggest that PepT2 plays an important role in the β‐Ala‐Lys‐AMCA uptake of BMECs.

### 3.5 Effect of PhT1 interference on β‐Ala‐Lys‐AMCA uptake

**FIGURE 7:**
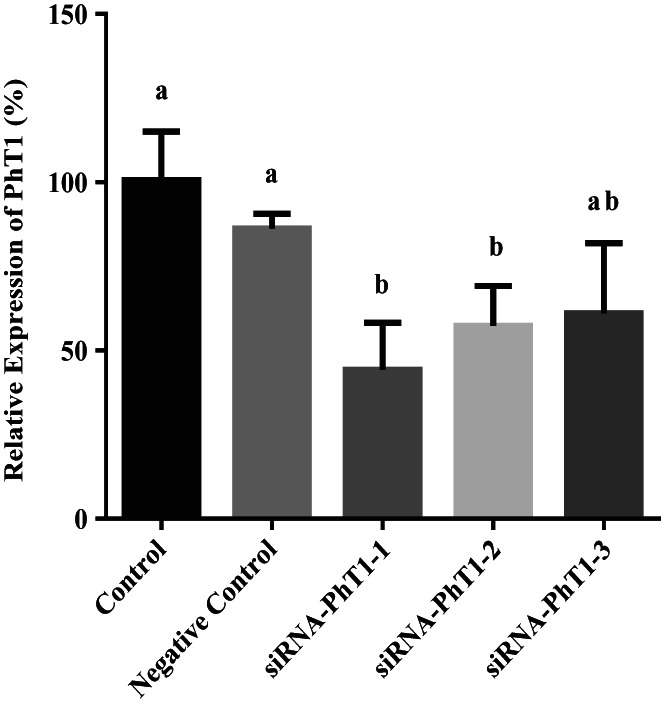
Effect of siRNA‐PhT1 on PhT1 mRNA abundance. The PhT1 mRNA abundance of siRNA‐PhT1s transfected groups (siRNA‐PhT1‐1, siRNA‐PhT1‐2, and siRNA‐PhT1‐3) and empty vectors transfected group (negative control) relative to nontransfected cells group (control) was calculated. Values are means ± SD (*n* = 3). Bars with different superscripts are significantly different (*p <* .05).

**FIGURE 8:**
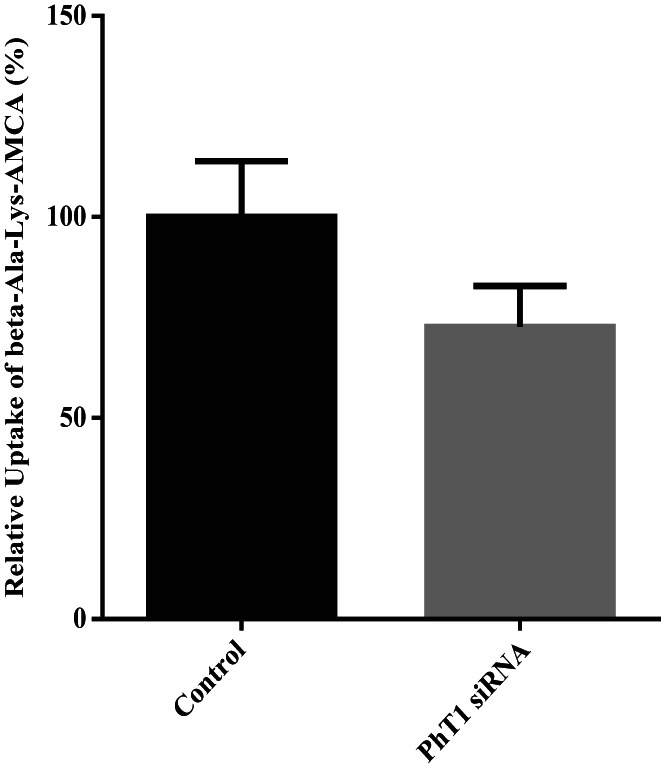
Effects of siRNA‐PhT1 on β‐Ala‐Lys‐AMCA uptake. The β‐Ala‐Lys‐AMCA uptake of siRNA‐PhT1 group relative to control group (nontransfected BMECs) was calculated. Values are means ± SD (*n* = 3).

The results showed that siRNA‐PhT1‐1 had the highest interference efficiency, which was 56%, and could be used in subsequent experiments (Figure 7). After transfection with siRNA‐PhT1‐1, the BMECs were incubated in lower chamber medium with 50 μM β‐Ala‐Lys‐AMCA at 37°C for 20 min. Then, the absorption of β‐Ala‐Lys‐AMCA by BMECs was detected. The results showed that after PhT1 interference, the uptake of β‐Ala‐Lys‐AMCA decreased by 27% (*p =* .051, Figure 8). These results suggest that PhT1 may also be involved in the uptake of β‐Ala‐Lys‐AMCA by BMECs.

## 4 DISCUSSION

Milk protein synthesis in the mammary gland is a complex process that is affected by many factors (Huang et al., 2021; Nichols et al., 2019; Omphalius et al., 2020). As substrates for milk protein, amino acids, and small peptides are the main factors affecting milk protein synthesis (Giallongo et al., 2016; Lean et al., 2018; Zhou et al., 2018). The results of some studies have shown that small peptides can regulate the synthesis of milk protein (Wang et al., 2018; Yang et al., 2015). However, the molecular mechanism of blood small peptide uptake by BMECs is still unclear. Some studies have shown that small peptides can promote the synthesis of milk protein in adherent cultured BMECs, and PepT2 plays an important role in the BMEC uptake of small peptides (Wang et al., 2018, 2019; Yang et al., 2015; Zhou et al., 2011, 2015). However, other studies have confirmed that PepT2 is expressed on the parietal membrane side of rat mammary epithelial cells and plays a role in the reabsorption of small peptides (milk protein hydrolysates and peptide‐like drugs) from milk, and the role of small peptides in promoting the expression of PepT2 and the synthesis of milk protein may occur in the parietal membrane side of BMECs (Shennan, 2016; Shennan & Boyd, 2014).

In this study, BMECs were cultured in a transwell chamber for the first time to study the mechanism of small peptide uptake in BMECs. The upper chamber represents the apical membrane side of epithelial cells (acinar cavity), and the lower chamber represents the basal membrane side of epithelial cells (blood). This barrier model is convenient for accurately studying the uptake mechanism of small peptides in BMECs. The results showed that BMECs were successfully cultured in transwells, and the cell barrier was successfully established, which could be used for the transmembrane transport test. Further experimental results showed that both upper and lower chambers Met‐Met uptake by BMECs increased the expression of β‐casein. The PepT2 was involved in the absorption of small peptides on both the apical and basal membrane sides of BMECs. A study by Wang et al. (2018) showed that PepT2 was expressed in both apical and basal membranes of BMECs, and it played an important role in the uptake of small peptides in the mammary gland of dairy cows. In addition, other studies have shown that PepT2 is expressed in the parietal membrane of rat mammary epithelial cells and plays a role in the process of reabsorption of small peptides from milk (Shennan, 2016; Shennan & Boyd, 2014). Our results are consistent with the above studies. In addition, the PhT1 may play a role in the uptake of small peptides in the basal membrane of BMECs. This is inconsistent with the results of Wang et al. (2018) and may be due to differences in cell culture methods.

## 5 CONCLUSION

The BMECs were successfully cultured in a transwell chamber and formed a cell layer. Both acinar and basal Met‐Met can be absorbed by BMECs through small peptide transporters and used for milk protein synthesis. Thus, the addition of small peptides in dairy cow diets may be an effective dietary manipulation to increase milk protein concentration or yield.

## CONFLICT OF INTEREST STATEMENT

The authors declare that they have no conflict of interest.

## ANIMAL WELFARE STATEMENT

The authors confirm that the ethical policies of the journal, as noted on the journal's author guidelines page, have been adhered to, and the appropriate ethical review committee approval has been received. The authors confirm that they have followed guidelines for the care and use of laboratory animals approved by the Special Committee on Scientific Research Ethics of Liaocheng University, Liaocheng, China (No. 2022112007).

## Data Availability

The data that support the findings of this study are available from the corresponding author upon reasonable request.
